# Comparative Study of Novel Ratio Spectra and Isoabsorptive Point Based Spectrophotometric Methods: Application on a Binary Mixture of Ascorbic Acid and Rutin

**DOI:** 10.1155/2016/2828647

**Published:** 2016-01-18

**Authors:** Hany W. Darwish, Ahmed H. Bakheit, Ibrahim A. Naguib

**Affiliations:** ^1^Department of Pharmaceutical Chemistry, College of Pharmacy, King Saud University, P.O. Box 2457, Riyadh 11451, Saudi Arabia; ^2^Department of Analytical Chemistry, Faculty of Pharmacy, Cairo University, Kasr El-Aini Street, Cairo 11562, Egypt; ^3^Pharmaceutical Analytical Chemistry Department, Faculty of Pharmacy, Beni-Suef University, Alshaheed Shehata Ahmad Hegazy Street, Beni-Suef 62514, Egypt

## Abstract

This paper presents novel methods for spectrophotometric determination of ascorbic acid (AA) in presence of rutin (RU) (coformulated drug) in their combined pharmaceutical formulation. The seven methods are ratio difference (RD), isoabsorptive_RD (Iso_RD), amplitude summation (A_Sum), isoabsorptive point, first derivative of the ratio spectra (^1^DD), mean centering (MCN), and ratio subtraction (RS). On the other hand, RU was determined directly by measuring the absorbance at 358 nm in addition to the two novel Iso_RD and A_Sum methods. The work introduced in this paper aims to compare these different methods, showing the advantages for each and making a comparison of analysis results. The calibration curve is linear over the concentration range of 4–50 *μ*g/mL for AA and RU. The results show the high performance of proposed methods for the analysis of the binary mixture. The optimum assay conditions were established and the proposed methods were successfully applied for the assay of the two drugs in laboratory prepared mixtures and combined pharmaceutical tablets with excellent recoveries. No interference was observed from common pharmaceutical additives.

## 1. Introduction

**Figure 1:**
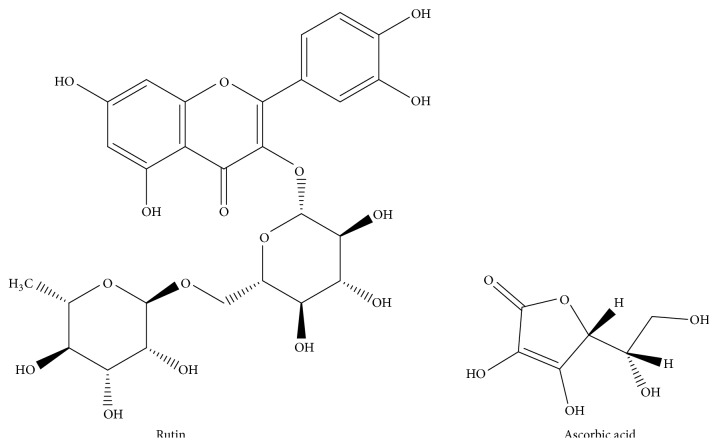
Chemical structures of rutin (RU) and ascorbic acid (AA).

Rutin (RU, Figure 1), chemically known as (quercetin-3-O-(6-O-rhamnosid) glucoside), is a well-known and available flavonoid present in many foodstuffs, such as buckwheat, onion, apple, tea, and red wine. RU is reported to possess several pharmacological properties. Studies have shown that RU has an antioxidant, anti-inflammatory, anti-carcinogenic, antimicrobial, and even antihypertensive activities. Additionally it works as an adjuvant for type 2 diabetes treatment [1–6]. RU is used in treating peripheral vascular disorders, due to its vascular protective property, for example, acute attack of piles, metrorrhagia, circulatory disturbances, and capillary fragility disorders [6]. Vitamin C (ascorbic acid) (AA, Figure 1) is chemically known as (2R)-2-[(1S)-1,2-dihydroxyethyl]-3,4-dihydroxy-2H-furan-5-one. The role of AA is a well-established endogenous antioxidant. AA is used principally for preventing and treating common cold [7]. Furthermore, supplements of AA are certified for treatment of certain respiratory disorders, including allergic rhinitis [8] and chronic rhino sinusitis [9]. Ruta C 60 tablets are available dosage form in the pharmaceutical market. It is composed of RU and AA. This combination is basically implemented for changing the enlarged fragility and penetrability of capillaries.

Different methods were reported for determination of AA and RU separately in drugs or other samples, but few were applied for simultaneous determination of AA and RU. These methods include UV-spectrophotometry [10], chemometric assisted spectrophotometry [11], electrochemical method [12], voltammetry [13], chemiluminescence [14], capillary electrophoresis [15–18], High Performance Liquid Chromatography (HPLC) [19–21], and near infrared spectroscopy (NIR) [22].

These methods lack simplicity due to intensive instrumentation used (e.g., HPLC and capillary electrophoresis) or some methods need complicated instrument and skilled analysts who can tackle sophisticated software (e.g., chemometric methods). The presented work introduces simple, rapid, selective, less expensive, and time saving procedures when compared to other previously published chromatographic methods. Additionally, these procedures have comparable precision and accuracy to the reported chemometric ones but with more simplicity. The simplicity is due to that there is no need for software as demanded by the latter [11]. This software is not familiar to every analyst which may hinder its wider application in solving analytical problems.

Hence, the first aim of the presented work is to introduce validated spectrophotometric procedures depending on manipulation of ratio spectra and isoabsorptive point for synchronized determination of RU and AA in pure powders, laboratory prepared mixtures, and pharmaceutical formulation. The second aim is to compare the results of the introduced procedures and show their merits (especially the new methods). AA was analyzed by seven methods while RU was analyzed by three methods.

## 2. Experimental

### 2.1. Apparatus

A double-beam UV-visible spectrophotometer (Shimadzu, Japan) model uv-1650 pc with quartz cell of 1 cm path length, connected to an IBM-compatible computer, was used.

The spectral bandwidth was 2 nm and wavelength-scanning speed 2800 nm/min. All recorded spectra converted to ASCII format by UV-probe personal spectroscopy software version 2.21.

### 2.2. Materials

All the chemicals used were of analytical grade, and the solvents were of HPLC grade. AA and RU (Sigma–Aldrich, USA), methanol (Chromasolv, for HPLC, Sigma–Aldrich) were used. Ruta C 60 tablets (Kahira Pharm. Chem. Ind. Co., Cairo, Egypt) are labeled to contain 60 mg of RU and 160 mg of AA (Batch number 1210864).

Stock solutions of RU (800 *μ*g/mL) and AA (800 *μ*g/mL) were readily prepared by dissolving 20 mg of RU and 20 mg of AA, individually in 25 mL methanol. Stock solutions were stable for at least three weeks when kept refrigerated at 4°C. Working solutions (200 *μ*g/mL) were prepared by suitable dilution in methanol.

Ruta C 60 tablets were weighed and finely powdered. An accurate weighed part of the powder corresponding to 60 mg of RU and 160 mg of AA was extracted twice into methanol utilizing sonication for 20 minutes and then the extract was filtered. The filtrate was diluted with methanol to final concentrations of 60 and 160 *μ*g/mL RU and AA, respectively. 500 *μ*L of Ruta C 60 tablet solution was transferred into a 5 mL measuring flask and diluted to mark with methanol to obtain final concentration of RU (6 *μ*g/mL) and AA (16 *μ*g/mL).

### 2.3. Construction of Calibration Curves

Portions corresponding to 40–500 *μ*g RU and 40–500 *μ*g AA were precisely taken from their standard working solutions (200 *μ*g/mL) into two separate sets of 10 mL measuring flasks; then the volume was completed with methanol. The spectra of the prepared standard solutions were scanned from 200 to 300 nm.

#### 2.3.1. Ratio Difference (RD) Method

For assaying of AA in presence of RU, the amplitude difference of the ratio spectra (AA/RU) at 240 and 330 nm (Δ*P*_240-330_) was plotted against the equivalent concentrations of AA in *μ*g/mL.

#### 2.3.2. Isoabsorptive_RD (Iso_RD) Method

The zero-order spectra of the prepared solutions were divided by the spectrum of 8 *μ*g/mL RU. The peak amplitudes of the ratio spectra were measured at 240 and 255 nm (isoabsorptive point). Calibration graphs were constructed relating the amplitudes at 255 nm (isoabsorptive point) and the differences in the amplitudes at 240 and 255 nm (Δ*P*_240-255_) to the corresponding concentrations of RU and AA, respectively.

#### 2.3.3. Amplitude Summation (A_Sum) Method

For quantitation of AA in presence of RU, the stored zero-order absorption spectra of RU and AA were derivatized in first order, using Δ*λ* = 4 and a scaling factor 10, and the first derivative amplitudes *D*_1_ were recorded. The amplitude factors of RU were calculated at the wavelengths [*D*_268_/*D*_381_]. The *D*_1_ amplitude of RU at 381 nm was multiplied by the previously calculated absorption factor [*D*_268_/*D*_381_] to obtain its amplitude at 268 nm; then the amplitude of RU and AA at 268 nm was plotted versus the equivalent concentrations of RU and AA in *μ*g/mL.

#### 2.3.4. ^1^DD Method

For quantitation of AA in presence of RU, the stored spectra of AA were divided by the spectrum of 8 *μ*g/mL RU and smoothed with Δ*λ* = 11 nm; then the first derivative of the ratio spectra (^1^DD) with Δ*λ* = 5 nm was attained. The amplitude of the first derivative peak of (AA/RU) was measured at 232 nm. A calibration graph was constructed relating the peak amplitude at 232 nm to the equivalent concentrations in *μ*g/mL of AA.

#### 2.3.5. Ratio Subtraction (RS) and Direct Methods

A calibration curve was constructed relating the absorbance of zero-order spectra of AA and RU at 246 nm and 358 nm, respectively, to their corresponding concentrations.

#### 2.3.6. Mean Centering (MCN) Method

The scanned spectra of AA were divided by the spectrum of 8 *μ*g/mL RU and the attained ratio spectra were smoothed with Δ*λ* = 11 nm and then mean centering step took place. A calibration curve was constructed by plotting the mean centered values at 241 nm for AA against the equivalent concentration.

#### 2.3.7. Isoabsorptive Point (Iso_P) Method

A calibration curve was constructed relating absorbance of the zero-order spectra of AA and RU at *λ* = 255 nm to their corresponding concentrations in *μ*g/mL and the regression equation was computed.

### 2.4. Assay of Synthetic Mixture

Different aliquots' ranges from 80.0 to 200.0 *μ*g of AA and 40–200 *μ*g of RU were transferred separately from their standard working solution (200 *μ*g/mL) into a set of 10 mL measuring flasks, completed to volume with methanol and mixed well. The spectra of the prepared solutions were recorded at 200–300 nm. The concentration of each analyte was determined by substitution in the equivalent regression equation after applying the corresponding manipulating steps for each method.

### 2.5. Analysis of Ruta C 60 Tablets

Ruta C 60 tablets were weighed and finely powdered. An accurately weighed portion of the powder equivalent to 60 mg of RU and 160 mg of AA was extracted twice into methanol with sonication for 20 minutes and then extract was filtered. The filtrate was diluted with methanol to get final concentrations of 60 and 160 *μ*g/mL for RU and AA, respectively. 500 *μ*L of Ruta C 60 tablet solution was transferred into a 5 mL volumetric flask and diluted to the mark with methanol to get a final concentration of RU (6 *μ*g/mL) and AA (16 *μ*g/mL). Spectral acquisition and the calculations were performed in the same manner as described in Section 2.3.

## 3. Results and Discussion

Ruta C 60 tablets are combined dosage form containing RU and AA. It is used for adjustment of increased fragility and permeability of capillaries. The ratio of AA : RU in their combined dosage form (Ruta C 60 tablets) is 8 : 3, respectively. The proposed work aims to construct simple, robust, and precise methods for simultaneous quantitation of AA and RU in Ruta C 60 tablets.

Molecular absorption spectroscopy is broadly applicable for quantitation of drugs in pharmaceutical dosage forms with increasing enhancements in analytical methodology. The implementation of this technique for analysis of pharmaceuticals has limitations that most active drugs absorb in the UV region and show severely overlapped spectra that make their simultaneous quantitation very difficult.

**Figure 2:**
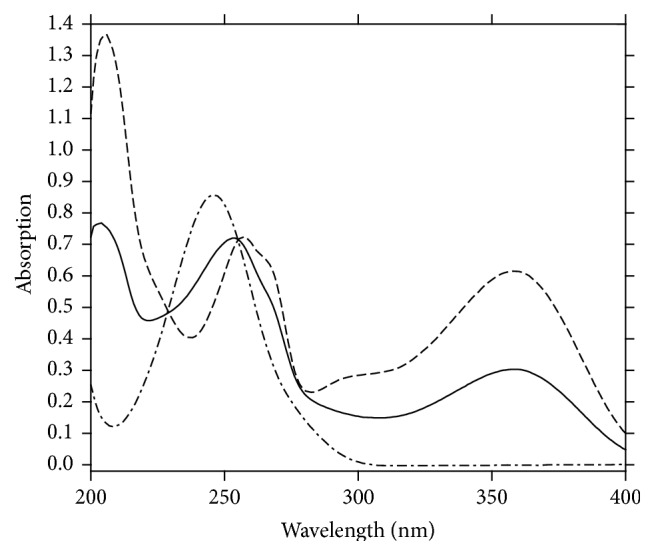
Zero-order absorption spectra of 8 *μ*g/mL AA (-·-·-·-), 8 *μ*g/mL RU (—), and a mixture of 4 *μ*g/mL AA with 4 *μ*g/mL RU (- - - - -) using methanol as blank.

In our introduced work, the zero-order absorption spectra (*D*_0_) of AA and RU exhibit overlapping as indicated in (Figure 2) which permits assay of RU in presence of AA at 358 nm, but hinders assay of AA in presence of RU directly. Accordingly, different methods depending on the isoabsorptive point concepts and manipulation of ratio spectra are introduced here for simultaneous quantitation of AA and RU in their synthetic mixtures as well as their combined dosage form.

### 3.1. Ratio Difference (RD) Method

Elzanfaly et al. [23–25] established a novel simple, fast, and selective procedure for simultaneous assay of drugs possessing interfering spectra in binary mixtures. The new procedure is characterized by lesser data processing as well as wider range of application when comparing with the previously published methods.

If we have drug *A* and drug *B* with interfering spectra, dividing the spectrum of *A* by a definite concentration of *B* leads to a ratio spectrum, where a direct relation between the amplitudes' difference (at any two wavelengths) and the equivalent concentration of *B* will be attained. The ratio spectrum of *B* will be represented by a straight line of constant amplitude parallel to the *x*-axis and the amplitudes' difference of *B* (at any two wavelengths) will equal zero. The mathematical explanation of the method could be described as follows.

In the ratio spectrum of a laboratory mixture of *A* and *B* divided by a divisor *B*′

(1) P1=P1A+K,P2=P2A+K,

where *P*_1_ and *P*_2_ represent the amplitudes at *λ*_1_ and *λ*_2_ of the mixture spectrum. *P*_1*A*_ and *P*_2*B*_ represent the amplitudes of *A* at *λ*_1_ and *λ*_2_, respectively. *K* represents the constant resulting from *B*/*B*′

(2) ΔPλ1-λ2P1−P2=P1A+K−P2A+K=P1X−P2X.

To determine *A* in the binary mixture samples, a calibration curve is constructed that relates the amplitudes' difference at *λ*_1_ and *λ*_2_ (Δ*P*_*λ*1-*λ*2_) in the ratio spectrum (utilizing divisor of definite concentration of *B*) to the equivalent concentration of *A*. Similarly, component *B* can be calculated utilizing divisor of definite concentration of *A*.

**Figure 3:**
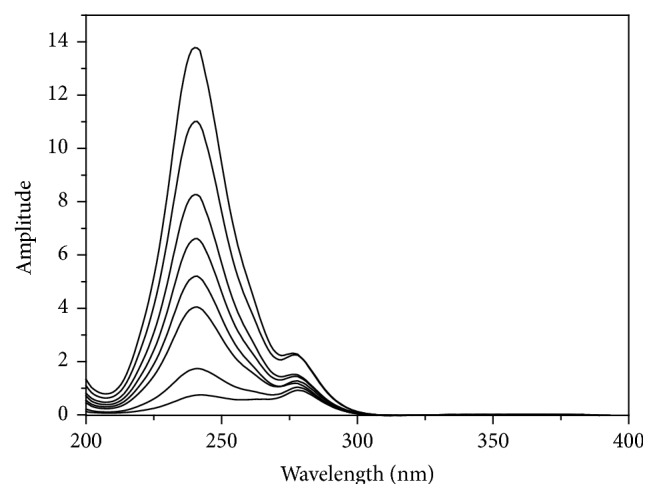
Smoothed ratio spectra of AA (4–50 *μ*g/mL) using 8 *μ*g/mL of RU as divisor and methanol as blank.

**Figure 4:**
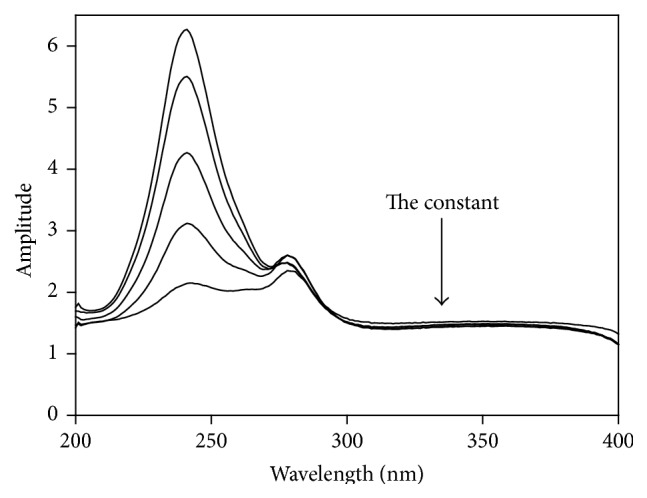
Ratio spectra of laboratory prepared mixtures of AA (*X*) and RU (*Y*) using 8 *μ*g/mL of RU (*Y*′) as a divisor and methanol as blank.

Accordingly, the initial step is the choosing of the divisors, where the selected divisors shall compromise between least noise and highest sensitivity. Various concentrations of divisor were utilized (e.g., 8, 40 *μ*g/mL and normalized spectrum) of RU, where divisor concentration of 8 *μ*g/mL of RU offered optimum accuracy, signal-to-noise ratio, repeatability, and sensitivity when applied to estimate AA concentrations in pure bulk powder and synthetic mixtures. Figures 3 and 4 show the smoothed ratio spectra of AA and that of synthetic mixtures of AA and RU, respectively, utilizing divisor of 8 *μ*g/mL of RU (*Y*′).

The next step is the selection of analytical wavelengths (at which measurements are recorded). Any two wavelengths can be selected as long as these two wavelengths show dissimilar amplitudes in the ratio spectrum and excellent linearity exists at each wavelength independently. Regarding AA, the wavelength pairs 240-228, 240-275, 240-290, 240-310, and 240-330 nm were applied; however 240-330 nm gave the best results.

Linear relation was attained between the differences in amplitude at 240-330 nm and the equivalent concentrations of AA. The regression equation was computed as follows:

(3) $$\begin{array}{c} \Delta P_{240.0\text{-}330.0}=0.285C+0.0332\;r=0.9964, \end{array}$$

where *C* represents the concentration of AA in *μ*g/mL, Δ*P* represents the amplitude difference at the designated wavelengths, and *r* is the correlation coefficient.

The main disadvantage of this method is the several trials to be done for selection of the proper devisor and the analytical wavelengths.

### 3.2. Isoabsorptive_RD (Iso_RD) Method

**Figure 5:**
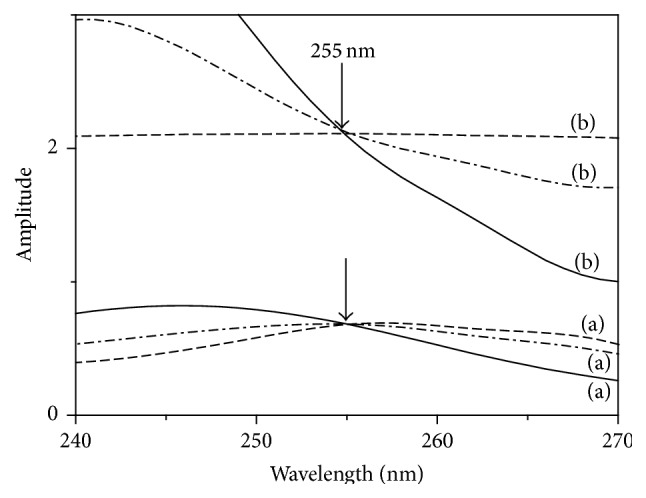
The spectra of 16 *μ*g/mL RU (- - - -), 16 *μ*g/mL AA (—), and a mixture containing 8 *μ*g/mL RU and 8 *μ*g/mL AA (-·-·-). (a) Absorption spectra in methanol. (b) Ratio spectra using a divisor of 8 *μ*g/mL RU in methanol as a blank.

The isoabsorptive point in the absorption spectrum of any given drug remains at the same wavelength in the ratio spectrum after division by a divisor of a given drug (as shown in Figure 5). Hence the total concentration of *A* and *B* can be calculated simultaneously using the regression equation that relates the isoabsorptive point amplitudes to the equivalent concentrations, in which drug *B* can be determined by subtraction. Isoabsorptive point in the ratio spectrum was combined with RD method (ISO_RD) for the simultaneous quantitation of AA and RU in their binary mixture.

Although RD method was solely able to determine the two drugs in two separate steps, the combination of the two methods decreased the number of steps needed to quantify the drugs, excluded the need for using two divisors (one for each drug), and decreased data handling.

The absorption spectra of AA show a degree of interference with that of RU as indicated in (Figure 2) that its assay in their binary mixture was not effective by applying direct spectrophotometric method.

As indicated above, thorough choice of divisor is critical, where best results were attained using divisor of 8 *μ*g/mL RU for the estimation of both drugs.

Various wavelengths were selected on the ratio spectra and the linearity at those wavelengths was evaluated. Best results and acceptable linearity at 240 for AA was attained, in addition to linearity at the isoabsorptive wavelength (255 nm) that was evaluated for the two drugs.

A linear relationship was constructed between the concentrations and the difference in amplitude of the ratio spectra at 240 and 255 nm for AA and at 255 nm for both AA and RU. Iso_RD is simpler than RD but still suffering from trails needed to be performed for choosing the appropriate devisor. Another limitation of this method is that the isoabsorptive point should exist, which may not always be the case.

### 3.3. Amplitude Summation (A_Sum) Method

A_Sum method is a new one that can be applied for a binary mixture of *X* and *Y*, where the spectra of *X* and *Y* exhibit isoabsorptive point at the zero spectrum, while first derivative spectra (*D*) of component *Y* shows no contribution with that of *X* at certain wavelength. After calculating the first derivative spectra (*D*) of equal concentration of *X* and *Y*, the *D* spectra intersect at isoabsorptive point at wavelength which shows certain shift from the isoabsorptive point found in zero-order spectra. Hence the following equation was obtained [26]:

(4) $$\begin{array}{c} D=\frac{dA}{d\lambda }=\frac{da}{d\lambda }bC. \end{array}$$

At *λ*_iso_ *D*_*X*_ = *D*_*Y*_

(5) $$\begin{array}{c} \frac{d_{X}}{d\lambda }b_{X}C_{X}=\frac{d_{Y}}{d\lambda }=b_{Y}C_{Y}. \end{array}$$

If *C*_*X*_ = *C*_*Y*_ and *b*_*X*_ = *b*_*Y*_, therefore

(6) $$\begin{array}{c} \frac{d_{X}}{d\lambda }=\frac{d_{Y}}{d\lambda }=\frac{d_{iso}}{d\lambda }. \end{array}$$

Therefore for a binary mixture, the amplitude at that wavelength (*λ*_iso_) can be calculated from the following equation:

(7) $$\begin{array}{c} D_{TM}=\frac{d_{iso}}{d\lambda }\left(\;C_{X}+C_{Y}\;\right), \end{array}$$

where *D*_TM_, *D*_*X*_, and *D*_*Y*_ are the *D* amplitudes of total mixture *X* and *Y*, respectively, at *λ*_iso_. *C*_*X*_ and *C*_*Y*_ are the concentrations of *X* and *Y*, respectively. *d*_*X*_ and *d*_*Y*_ are the differences of the absorptivities of *X* and *Y* at the specified *dλ*. *b*_*X*_ and *b*_*Y*_ are the path lengths (1 cm). Proving that *X* and *Y* interfere at *λ*_iso_ and *X* does not exhibit any interference at wavelength *λ*_1_; hence the postulated amplitude of *Y* in the mixture at *λ*_iso_ can be calculated using its response factor [26] between the two proposed wavelengths (*λ*_1_ and *λ*_iso_). We can calculate the amplitude corresponding to *X* by difference between recorded amplitude corresponding to total (*X* + *Y*) and the postulated amplitude corresponding to *Y*. Concentrations of *X* and *Y* can be determined using the same regression equation (attained by plotting the first derivative amplitude (*D*) of either *X* or *Y* at *λ*_iso_ against their corresponding concentrations).

**Figure 6:**
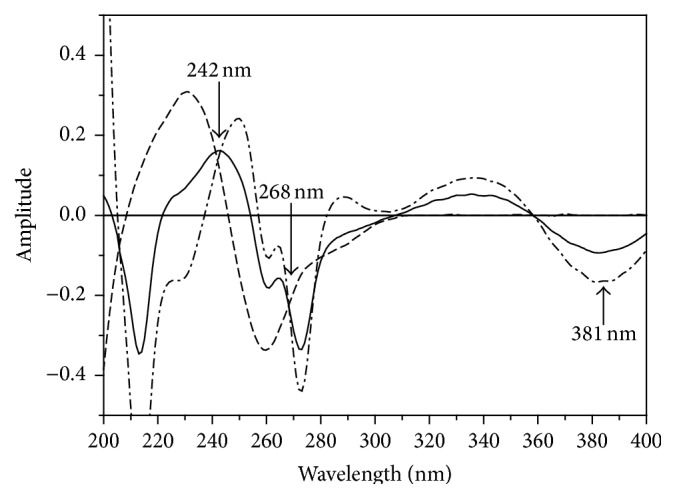
First derivative spectra of 8 *μ*g/mL AA (- - - - -), 8 *μ*g/mL RU (-·-·-·-), and a mixture of 4 *μ*g/mL AA with 4 *μ*g/mL RU (—) using methanol as blank.

Accordingly, the isoabsorptive point in derivative absorption spectra of AA and RU binary mixture was determined (at 268 nm as shown in Figure 6). The ^1^D amplitude of RU at 268 nm was calculated using its amplitude response factor between 268 nm and 381 nm, and then the corresponding amplitude of AA was obtained by subtraction. The ^1^D amplitude of AA and RU was used to calculate their corresponding concentration using the unified regression equation at *λ*_iso_ 281 nm.

 
*D*_RU_ at 268 nm = [*D*_268_/*D*_381_]. (*D*_mix_ at 381 nm),
 
*D*_AA_ at 268 nm = *D*_mix_ at 268 nm – *D*_RU_ at 268 nm,

where *D*_mix_ is the ^1^D amplitude value of the binary mixture; *D*_AA_ and *D*_RU_ are the ^1^D amplitudes of AA and RU, respectively; and [*D*_268_/*D*_381_] is the ratio of the ^1^D amplitudes of pure RU at 268 nm to that at 381 nm.

The leading benefit of A_Sum method is that there is no need for a divisor to apply the derivative technique; however absence of isoabsorptive point may hinder its application on mixtures lacking this point in their spectra.

### 3.4. ^1^DD Method

Salinas et al. [27] proposed a spectrophotometric method depended on derivation of the ratio spectra for analyzing binary mixtures. The principal benefit of the adopted ratio-spectra derivative spectrophotometric method is the possibility of running easily measurements in correspondence of peaks so it allows using wavelength of maximum intensity (a maximum or a minimum) [28–30]. Furthermore, the existence of a number of maxima and minima is another benefit as it permits the quantitation of active drugs in presence of other drugs and additives which probably intervene with the assay. In the proposed procedure the absorption spectrum of the mixture (absorbance at every wavelength) is divided by the absorption spectrum of a reference standard solution of one of the drugs. Then the first derivative of the ratio spectrum is acquired and the concentration of the other drug is then calculated from a calibration graph.

**Figure 7:**
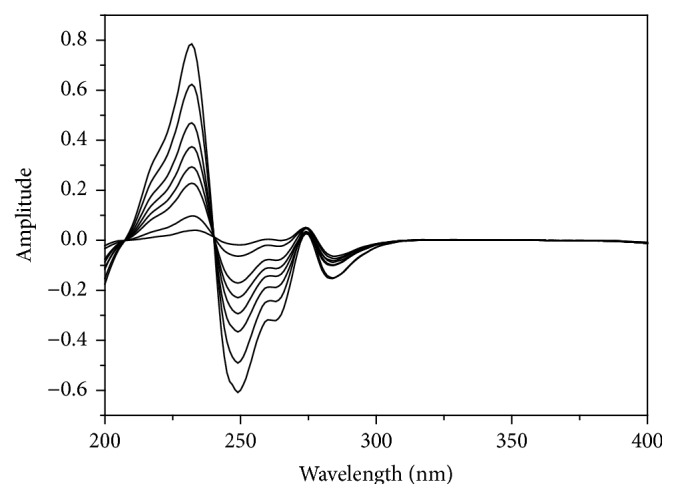
First derivative of smoothed ratio of AA (4–50 *μ*g/mL) using 8 *μ*g/mL of RU as a divisor and methanol as blank.

The main factors that influence the shape of the ratio spectra were carefully studied and optimized, which include speed of scanning, divisor's concentration, wavelength increase over which the derivative is attained (Δ*λ*), and the smoothing function. The ratio spectra and the first derivative of the ratio spectra are showed in Figures 3 and 7, respectively. Regarding the influence of wavelength scanning speed, it was found that, at great speed, noisy spectra are attained while at low scanning speed, the noise is reduced but a longer time is required for the measurements; hence medium scanning speed was selected to complete measurements. The influence of divisor concentration was tested as well, and the best average recovery percent was achieved using divisor of 8 *μ*g/mL of RU. Optimization of all these factors may need effort, which is considered disadvantage of this method. On the other hand, the independence of ^1^DD method of the existence of isoabsorptive point provides it wider application than the isoabsorptive point based methods.

The absorption spectra of AA were divided by the absorption spectrum of 8 *μ*g/mL RU and smoothed (Figure 3).  Figure 7 shows the equivalent first derivative of the ratio spectra of Figure 3. For calibration graph, the wavelengths were selected that demonstrated the fittest linear relationship to the concentration of AA (232 and 250 nm). Measuring the amplitude between 232 and 250 nm did not display significant enhancement in the recovery percent. The peak amplitudes of the first derivative of ratio spectra were then recorded at 232 nm. Good linearity was attained in the concentration range of 4.0–50.0 *μ*g/mL for AA. The linear regression equation is found to be

(8) $$\begin{array}{c} P_{AA}=0.0161C+0.0332\;r=0.9964, \end{array}$$

where *C* is the concentration of AA in *μ*g/mL, *P* is the peak amplitude of the first derivative of the ratio spectrum curve, and *r* is the correlation coefficient.

### 3.5. Ratio Subtraction (RS) and Direct Methods

**Figure 8:**
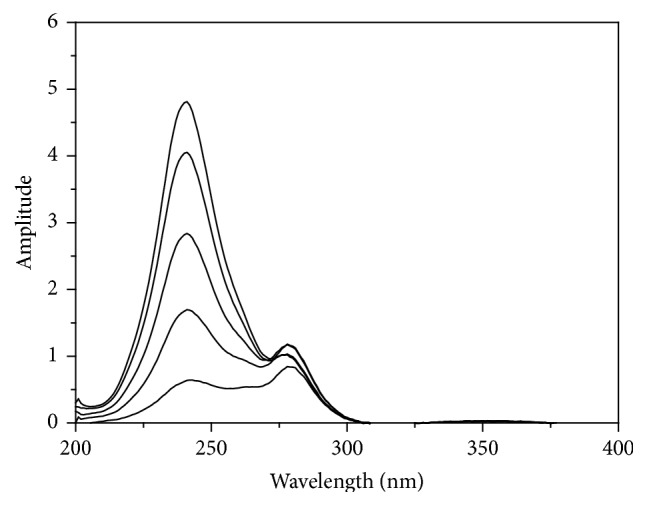
Ratio spectra of laboratory prepared mixtures of AA (*X*) and RU (*Y*) using 8 *μ*g/mL of RU (*Y*′) as a divisor and methanol as a blank after subtraction of the constant.

**Figure 9:**
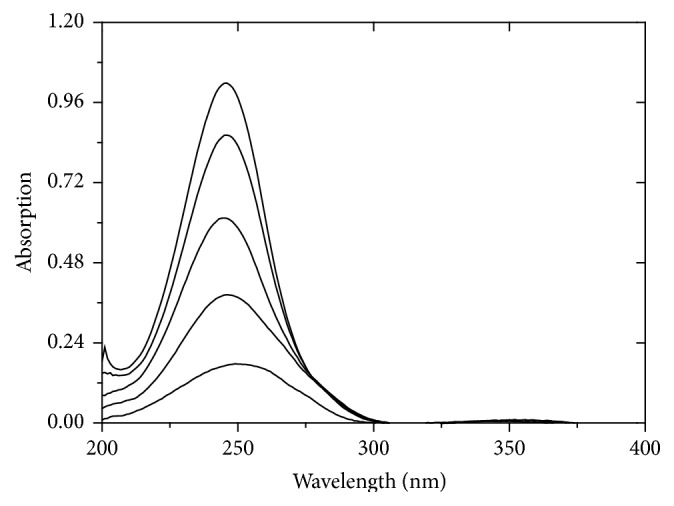
The zero-order absorption spectra of AA obtained by the proposed ratio subtraction method for the analysis of laboratory prepared mixtures after multiplication by the divisor (*Y*′).

The ratio subtraction method [31] began by scanning zero-order spectra of the methanolic standard solutions of AA; then the linearity was evaluated between absorbance at the selected wavelength 246 nm and the corresponding concentration of AA. The method principle depends on that, for a mixture of *X* (AA) and *Y* (RU) and the spectrum of (*Y*) is more extended as shown in Figure 2; assay of *X* can be achieved by scanning the zero-order absorption spectra of the synthetic mixtures (AA and RU) and then dividing them by sensibly selected concentration (8 *μ*g/mL) of standard RU (*Y*′ = divisor) giving new ratio spectra that represent *X*/*Y*′ +  constant  as revealed in Figure 4. Furthermore, subtraction of absorbance values of these constants (*Y*/*Y*′) was done in plateau region as displayed in Figure 8, followed by multiplication of the acquired spectra by (*Y*′), the divisor, as shown in Figure 9. Lastly, the original spectra of (*X*) could be gotten which were used for direct assay of AA at 246 nm utilizing the corresponding regression equation for determination of AA concentrations. This method could be the method of choice when there is an extended part of the spectrum of one of the analytes but also may represent a major limitation for this method when this condition is lost.

A linear relationship was attained between the absorbance and the equivalent concentration of AA at 246 nm. The regression equation is

(9) $$\begin{array}{c} P_{AA}=0.6381C+0.0241\;r=0.9964, \end{array}$$

where *C* represents the concentration of AA in *μ*g/mL, *P* represents the peak amplitude of the zero-order spectrum of AA at 246 nm, and *r* represents the correlation coefficient.

However, for determination of RU alone, a calibration curve was constructed relating the absorbance of zero-order spectra of RU at 358 nm, where AA shows no absorbance (Figure 2), to the corresponding concentrations, and the regression equation is

(10) $$\begin{array}{c} A_{RU}=0.0385C+0.0206\;r=0.9999, \end{array}$$

where *C* represents the concentration of RU in *μ*g/mL, *A* represents the absorbance of RU at 358 nm, and *r* represents the correlation coefficient.

### 3.6. Mean Centering (MCN) Method

For additional enhancement of selectivity to resolve the interference existing between AA and RU, a simple method that depended on mean centering [32, 33] of ratio spectra was adopted. It removes the step of derivative calculation and hence the signal-to-noise ratio was greatly improved [32, 33] and this was clear from the low value of LOD (limit of detection) of AA using MCN method (Table 5).

Basic principle for the mean centering method is illustrated fully in literature [34].

**Figure 10:**
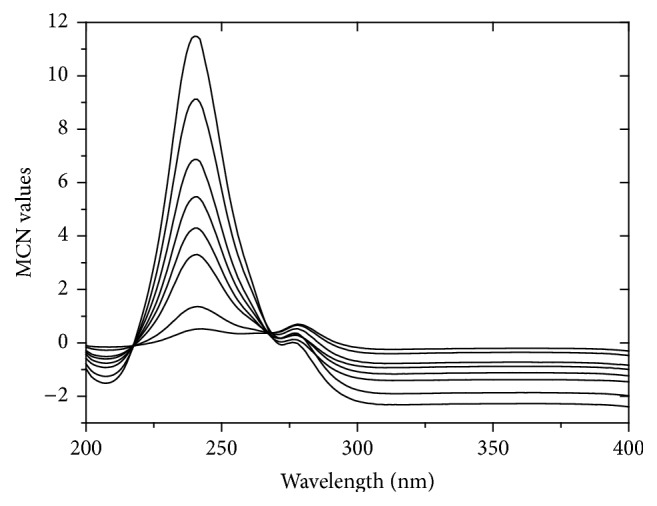
Mean centered ratio spectra of AA (4–50 *μ*g/mL) using 8 *μ*g/mL of RU as a divisor and methanol as blank.

Mean centering method was applied to assay AA quantitatively in presence of RU in their synthetic mixtures and in their combined dosage form. The absorption spectra of AA were divided by the absorption spectrum of 8 *μ*g/mL RU followed by smoothing procedure (Figure 3). The acquired ratio spectra were then mean centered and the concentrations of AA were calculated by measuring the amplitude at 241 nm (Figure 10). The linear regression equation is computed:

(11) $$\begin{array}{c} MCN_{AA}=4.6640C+1.0217\;r=0.9966, \end{array}$$

where *C* represents the concentration of AA in *μ*g/mL, MCN_AA_ represents the peak amplitude of the mean centered ratio spectrum curve, and *r* represents the correlation coefficient.

### 3.7. Isoabsorptive Point (Iso_P) Method

For AA quantitation, isoabsorptive method [31] was applied for estimation of total concentration of AA and RU. Absorption spectra of 8 *μ*g/mL of AA, of 8 *μ*g/mL of RU, and of a mixture containing 4 *μ*g/mL of each of AA and RU displayed isoabsorptive point at 255 nm (Figure 2). By measuring absorbance at selected isoabsorptive point in the absorption spectrum, the total concentration of AA and RU in the mixture could be determined and consequently AA concentration was calculated by subtraction of RU concentrations.

The total concentration of AA and RU could be calculated using the following equation:

(12) $$\begin{array}{c} A_{255}=0.5061C+0.0208\;\left(r=0.9955\right), \end{array}$$

where *C* represents the concentration of total concentration of AA and RU in *μ*g/mL, *A* represents the absorbance of AA or RU at 255 nm, and *r* represents the correlation coefficient. The major limitation of this method is that it needs a complementary analytical method for determination of one of the analytes in the mixture.

The selectivity of the adopted methods was evaluated by the analysis of synthetic mixtures containing various ratios of AA and RU, where adequate results were attained over the calibration ranges as abridged in Table 1. Additionally, the suggested methods were applied for assay of the two drugs in their combined dosage form (Table 2).

Results attained by the proposed methods for assay of AA and RU in Ruta C 60 tablets were compared statistically to the results attained by the reported PLS method [11].

The results indicated no significant differences between the suggested methods and the reported one as displayed in Table 3. Furthermore, ANOVA test was performed (Table 4), demonstrating that there is no significant difference between all proposed spectrophotometric methods. Additionally, a validation sheet is anticipated in Table 5.

## 4. Methods Validation

### 4.1. Linearity and Sensitivity

Methods' linearity was assessed by analyzing different concentrations of AA and of RU ranging between 4–50 *μ*g/mL and 4–40 *μ*g/mL, respectively. Each concentration was repeated three times. The assay was done according to the previously mentioned experimental conditions (Table 5).

Calculation of limit of quantitation (LOQ) and limit of detection (LOD) was competed according to the ICH Q2 (R1) recommendation [35]. The limit of quantitation (LOQ) was calculated by determining the lowest measurable concentration, below which the calibration graph becomes nonlinear. The limit of detection (LOD) was calculated by determining the lowest concentration of analytes that can be detected. The values of LOQ and LOD were computed according to the next equations:

(13) LOD=3.3σS,LOQ=10σS,

where *σ* represents standard deviation of the intercept of regression line and *S* represents slope of regression line of calibration curve. The results are given in Table 5.

### 4.2. Accuracy

Evaluation of accuracy of the results was evaluated by applying the suggested methods for assay of various blind samples of AA and RU. The concentrations were attained from the equivalent regression equations; then percentage recoveries were computed with mean percentage recovery shown in Table 5.

### 4.3. Selectivity

Evaluation of methods' selectivity was attained by assay of various synthetic mixtures of AA and RU within their calibration ranges. Adequate results are anticipated in Table 1. Additionally there was no interference found from additives found in their combined dosage form as indicated in Table 2.

### 4.4. Range

Calibration range was obtained through considerations of the practical range necessary according to obedience to Beer's law and the concentration of AA and RU existed in the dosage form. The results are displayed in Table 5.

### 4.5. Precision

#### 4.5.1. Repeatability

Three different concentrations of AA and RU (12, 16, and 20 *μ*g/mL) were assayed three times intradaily by applying the suggested methods. The percentage recoveries and relative standard deviation were computed as abridged in Table 5.

#### 4.5.2. Reproducibility (Intermediate Precision)

The above-mentioned procedures were repeated interdaily on three various days for assaying the three selected concentrations. The percentage recoveries and relative standard deviation were computed as shown in Table 5.

### 4.6. Stability

AA and RU working solution showed no spectrophotometric changes for not less than 21 days when kept at 4°C.

## 5. Conclusions 

This work discussed the analysis of binary mixture of RU and AA without prior separation depending on the manipulation of ratio spectra and isoabsorptive point. The work included novel spectrophotometric methods such as ratio difference, isoabsorptive-ratio difference, and amplitude summation methods. These methods are characterized by their minimal data analysis procedures as there is no need for complementary method as in the case of the traditional isoabsorptive point method. The results were statistically comparable to the reported PLS method. Finally, it can be concluded that the suggested methods are simple and do not need sophisticated techniques or instruments. Additionally, they are sensitive and selective and could be applied for routine analysis of AA and RU in their combined dosage form. Furthermore, the methods are appropriate and valid for application in laboratories lacking liquid chromatographic instruments.

## Figures and Tables

**Table 1:** Determination of AA and RU in laboratory prepared mixtures by the proposed spectrophotometric methods.

Concentration		Direct	A_Sum	Iso_RD	A_Sum	Iso_RD	Iso_P	RD	^1^DD	MCN	RS
(*µ*g/mL)		*R*%^a^	*R*%^a^	*R*%^a^	*R*%^a^	*R*%^a^	*R*%^a^	*R*%^a^	*R*%^a^	*R*%^a^	*R*%^a^
AA	RU	RU	RU	RU	AA	AA	AA	AA	AA	AA	AA
8	4	104.69	99.40	100.13	101.22	101.06	91.20	106.37	102.40	104.64	102.84
8	8	105.01	99.50	97.78	99.24	99.75	98.71	104.74	103.43	103.86	103.47
12	8	99.18	100.48	101.62	99.72	98.49	98.48	101.91	100.21	99.99	101.32
8	12	103.75	98.67	99.36	98.64	99.75	96.20	101.58	100.12	101.00	100.49
12	12	99.60	99.12	102.50	98.95	98.41	96.93	101.36	99.91	100.61	100.75
16	12	98.63	100.16	97.77	101.26	100.75	91.02	99.04	98.02	99.58	98.09
20	12	99.94	99.35	102.37	101.20	101.79	90.86	99.39	98.48	100.21	98.68
8	16	106.27	98.91	98.53	99.10	95.94	97.47	103.69	102.69	103.84	102.56
12	16	101.07	101.57	96.02	98.80	100.11	96.03	102.66	102.07	103.03	101.77
20	16	100.31	99.06	97.19	99.92	97.34	96.12	103.34	102.77	104.19	102.93
8	20	106.58	99.55	99.12	99.35	102.21	96.33	101.45	101.15	101.51	100.54
20	20	99.18	101.56	99.72	100.35	100.13	96.94	103.40	102.56	104.00	102.76

Mean		102.02	99.78	99.34	99.81	99.65	95.52	102.41	101.15	102.21	101.35

SD		2.88	0.97	2.05	0.98	1.83	2.73	2.00	1.71	1.81	1.64

RSD%		2.82	1.21	2.06	0.98	1.84	2.86	1.95	1.69	1.77	1.62

^a^Average of four determinations.

**Table 2:** Determination of AA and RU in tablets (Ruta C 60) batch number 1210864.

Method		Direct	A_Sum	Iso_RD	A_Sum	Iso_RD	Iso_P	RD	^1^DD	MCN	RS
RU	AA	RU	RU	RU	AA	AA	AA	AA	AA	AA	AA
Labelled conc. (*μ*g mL^−1^)		*R*%^a^	*R*%^a^	*R*%^a^	*R*%^a^	*R*%^a^	*R*%^a^	*R*%^a^	*R*%^a^	*R*%^a^	*R*%^a^
6	16	102.94	101.88	100.24	100.40	100.14	101.21	102.59	101.21	101.34	100.85
6	16	100.67	100.31	98.75	101.39	98.80	98.89	100.71	98.89	99.30	98.87
6	16	98.05	95.98	102.25	100.13	98.80	95.82	97.51	95.82	96.18	95.82
6	16	98.27	98.47	102.29	102.11	97.37	97.69	98.83	97.69	97.69	96.90
6	16	99.51	99.18	102.94	101.94	97.36	97.48	99.50	97.48	98.18	97.62

Mean (%)		99.89	99.16	101.29	101.19	98.50	99.34	98.22	98.22	98.54	98.01

SD		2.00	2.20	1.74	0.89	1.17	2.13	2.00	2.00	1.93	1.94

^a^Average of four determinations.

**Table 3:** Statistical comparison for the results obtained by the proposed spectrophotometric methods and the reported method [11]^a^ for the analysis of AA and RU (Ruta C 60) batch number 1210864.

Value	Direct	A_Sum	Iso_RD	A_Sum	Iso_RD	Iso_P	RD	^1^DD	MCN	RS	Reported PLS method	
	RU	RU	RU	AA	AA	AA	AA	AA	AA	AA	RU	AA
Mean^b^	97.62	99.16	101.29	101.19	98.49	99.38	99.83	98.22	98.54	98.01	100.03	100.40

SD	1.61	2.20	1.75	0.89	1.17	2.03	1.93	2.00	1.93	1.94	3.34	1.92

RSD%	2.00	2.22	1.72	0.88	1.19	2.14	1.93	2.04	1.96	1.98	3.34	1.91

*N*	5	5	5	5	5	5	5	5	5	5	5	5

Variance	2.58	4.83	3.05	0.80	1.36	4.35	3.65	3.99	3.70	3.75	11.14	3.68

*F* value^b^	4.323 (6.388)	2.307 (6.388)	3.649 (6.388)	4.605 (6.388)	2.703 (6.388)	1.119 (6.388)	1.086 (6.388)	1.011 (6.388)	1.008 (6.388)	1.018 (6.388)		

*t*-test^b^	1.453 (2.447)	0.482 (2.365)	0.752 (2.447)	1.943 (2.447)	1.902 (2.365)	0.820 (2.306)	0.474 (2.306)	1.765 (2.306)	1.535 (2.306)	1.963 (2.306)		

^a^PLS method.

^b^The values in the parenthesis are the corresponding theoretical values of *t* and *F* at *p* = 0.05.

**Table 4:** One-way ANOVA testing for the different proposed methods used for the determination of AA and RU in Ruta C 60 tablets (batch number 1210864).

	Source of variation	DF	Sum of squares	Mean square	*F* value
AA	Between exp. Within exp.	6 28	40.662 85.305	6.777 3.047	2.224

RU	Between exp. Within exp.	2 12	11.730 47.573	5.865 3.964	1.479

There was no significant difference between the methods using one-way ANOVA (*F*-test), where *F* tabulated = 2.445 for AA and 3.885 for RU and at *p* < 0.05.

**Table 5:** Assay validation sheet of the proposed spectrophotometric methods for the determination of AA and RU.

Parameter	Direct	A_Sum	Iso_RD	A_Sum	Iso_RD	Iso_P	RD	^1^DD	MCN	RS
	RU	RU	RU	AA	AA	AA	AA	AA	AA	AA
Accuracy										
(Mean ± SD)	99.91 ± 0.35	96.99 ± 0.55	100.44 ± 1.11	99.66 ± 1.00	101.13 ± 1.42	101.36 ± 1.74	102.10 ± 0.88	101.54 ± 0.24	101.95 ± 0.63	99.46 ± 1.55

Precision										
Repeatability^a^	100.28 ± 0.95	95.79 ± 0.63	99.93 ± 1.79	99.93 ± 0.90	99.42 ± 2.02	98.11 ± 1.29	101.36 ± 1.25	99.86 ± 1.29	101.57 ± 1.29	100.49 ± 1.27
Intermediate precision^b^	100.86 ± 2.15	97.91 ± 1.31	98.60 ± 1.32	99.30 ± 1.56	98.09 ± 1.97	99.80 ± 2.17	100.91 ± 1.69	100.75 ± 1.49	100.98 ± 1.32	100.55 ± 1.67

Linearity										
Slope	0.0385	−0.0017	0.1416	−0.0017	0.1480	0.5061	0.2850	0.0161	4.6640	0.6381
Intercept	0.0206	0.0042	−0.3615	0.0042	−0.4926	0.0208	−0.0332	0.0332	1.0217	0.0241
Correlation coefficient (*r*)	0.9999	−0.9999	1	−0.9999	0.9952	0.9955	0.9964	0.9964	0.9966	0.9964
Range (*μ*g mL^−1^)	4–50	4–50	4–50	4–50	4–50	4–50	4–50	4–50	4–50	4–50

LOQ (*μ*g mL^−1^)	1.13	1.39	0.49	1.39	1.54	3.04	1.20	0.42	1.04	2.70

LOD (*μ*g mL^−1^)	0.37	0.46	0.16	0.46	0.51	1.00	0.40	1.28	0.34	0.89

^a^The intraday (*n* = 3), average of three concentrations (12, 16, and 20 *μ*g mL^−1^) for AA and RU repeated three times within the day.

^b^The interday (*n* = 3), average of three concentrations (12, 16, and 20 *μ*g mL^−1^) for AA and RU repeated three times in three successive days.
